# Puribacter membranae gen. nov., sp. nov., isolated from a biofilm of a membrane bioreactor (MBR) treating sewage

**DOI:** 10.1099/ijsem.0.006978

**Published:** 2025-11-20

**Authors:** Masashi Hatamoto

**Affiliations:** 1 Department of Civil and Environmental Engineering, Nagaoka University of Technology, Niigata 940-2188, Japan

**Keywords:** activated sludge, biofilm, *Burkholderiaceae*, g_CTSOIL-112

## Abstract

Novel Gram-stain-negative, non-spore-forming, non-motile rods, designated HTMS2 and HTMS3^T^, were isolated from a biofilm on the membrane of a municipal sewage treatment membrane bioreactor in Nagaoka, Japan. Phylogenetic analysis of 16S rRNA genes placed them in the family *Burkholderiaceae*, most closely related to *Hydromonas duriensis* A2P5 ^T^ (94.14% similarity). Genome sequencing (2.52 Mb, 48.2% G+C) and phylogenomic analysis affiliated them with the uncultured genus lineage CTSOIL-112 in the Genome Taxonomy Database. Average nucleotide identity (ANI) and digital DNA–DNA hybridization (dDDH) values to related taxa were below species thresholds (ANI ≤77.0%, dDDH ≤54.9%). In addition, average amino acid identity values to related taxa were ≤68.6%. Both strains are catalase- and oxidase-positive, reduced nitrate and utilized various carbohydrates but not *N*-acetyl-glucosamine; they differed in sorbitol assimilation. The major respiratory quinone is Q-8, and the predominant fatty acids are summed feature 3 (C_16:1 ω7c/ω6c), summed feature 8 (C_18:1 ω7c/ω6c) and C_16:0. Genomic data supported a non-motile, Gram-stain-negative phenotype. Polyphasic analysis indicates that these strains represent a novel genus and species, *Puribacter membranae* gen. nov., sp. nov. (type strain HTMS3^T^=NBRC 117344^T^=LMG 34133^T^).

## Introduction

Membrane bioreactors (MBRs) have become widely used in recent wastewater treatment plants because they efficiently produce high-quality effluents by combining biological treatment with membrane filtration [1]. Despite their advantages, a significant operational challenge is membrane fouling mainly caused by the formation of microbial biofilms on the membrane surface, which reduces treatment efficiency and increases operational costs [2,3]. Biofilm formation is driven by complex microbial communities, and identifying the key bacterial components within these biofilms is crucial for understanding fouling mechanisms and developing effective control strategies [2,4].

The microbial communities within MBR biofilms are highly diverse and include various bacteria belonging to the family *Burkholderiaceae*, which have a wide ecological distribution and metabolic versatility. Members of *Burkholderiaceae* have been isolated from a range of habitats, including soil, freshwater, marine environments, plant tissues and engineered systems such as wastewater treatment reactors. In recent years, several novel genera have been described within this family, such as *Zeimonas* [5], *Imbroritus* [6], *Formosimonas* [7], *Hydromonas* [8] and *Ephemeroptericola* [9], highlighting its significant taxonomic diversity. As a result of these intensive studies, the family comprised 26 genera with validly published names according to the LPSN (https://www.bacterio.net/index.html) at the time of writing.

During an investigation of microbial biofilms collected from membrane surfaces in a municipal sewage treatment MBR system, two closely related Gram-stain-negative bacterial strains were isolated, designated strains HTMS2 and HTMS3^T^. Preliminary analyses indicated that these strains represented an uncharacterized genus-level lineage, named CTSOIL-112 based on Genome Taxonomy Database (GTDB) [10] within the family *Burkholderiaceae*. In this study, the polyphasic taxonomic characterization of these strains is described, and the novel genus and species *Puribacter membranae* gen. nov., sp. nov. are proposed.

## Methods

### Isolation and culture conditions

Biofilm samples were collected from the membrane surface of an experimental MBR installed at the Nagaoka Central Sewage Treatment Center, Nagaoka, Japan [11]. The biofilm sample was diluted with ultrapure water, spread onto LB agar plates [2% (w/v) LB medium containing 20 g l^−1^ tryptone, 10 g l^−1^ yeast extract, 5 g l^−1^ NaCl and 1.5% (w/v) agar] and incubated at 37 °C. The strains were purified by repeatedly streaking single colonies onto LB agar plates.

### 16S rRNA phylogenetic analysis

Total genomic DNA was extracted using the Kaneka Easy DNA Extraction Kit version 2 (KANEKA, Japan), based on the manufacturer’s instructions. PCR amplification of the 16S rRNA gene was performed using the Back 8F mix (5′-AGA GTT TGA TYM TGG CTC AG-3′) and Univ. 1492 rm (5′-GGGH TAC CTT GTT ACG ACT T-3′) [12]. The PCR product was purified using the QIAquick PCR Purification Kit (QIAGEN, Germany) and subjected to Sanger sequencing ordered for Fasmac Co., Ltd. (Atsugi, Japan) after its quality was confirmed using agarose gel electrophoresis. The taxonomic classification of the strains was determined by comparing their 16S rRNA gene sequences with 16S rRNA from the Bacteria and Archaea type strains database of the National Center for Biotechnology Information (NCBI) using Basic Local Alignment Search Tool, and collecting the sequence information of their relative strains. Phylogenetic trees based on the 16S rRNA sequences of strains HTMS2 and HTMS3^T^ and related type strains of members of the family *Burkholderiaceae* were reconstructed using the neighbour-joining, maximum-likelihood and maximum-parsimony methods with the mega 12 software package [13]. The robustness of the phylogenetic trees was evaluated using 1,000 bootstrap resampling analyses [14].

### Physiology and chemotaxonomy analysis

Morphological characteristics were determined using cultures grown on modified LB agar (per litre: 5 g of tryptone, 2.5 g of yeast extract, 1 g of NaCl and 15 g of agar) at 30 °C for 48 h under aerobic conditions. Cell morphology and size were examined using optical microscopy (BX50F4, Olympus, Japan). Colony morphology was observed under the same growth conditions. Gram staining was performed using Favor-G Nissui (Nissui Pharmaceutical, Japan). Physiological characteristics, including catalase and oxidase reactions, acid or gas production from glucose fermentation and oxidation-fermentation tests of glucose, were performed following the standard procedures [15]. Biochemical properties were determined using API 20 NE, API 20 E, API 50 CH and API ZYM kits (bioMérieux, France), according to the manufacturer’s instructions. All tests were conducted at Techno Suruga Laboratory Co., Ltd. (Shizuoka, Japan).

The optimal pH for the strain was determined by varying pH (4–10 in intervals of 1.0 pH units) with LB medium (Lennox, Difco). A medium was used with appropriate buffer systems: 0.5 M Tris-HCl (pH 8–10), 0.5 M MOPS (pH 7), 0.5 M MES (pH 5–6) and 0.1 M citrate buffer (pH 4). The effects of temperature (4 °C, 10 °C, 15 °C, 20 °C, 28 °C, 37 °C, 45 °C and 50 °C) on the growth of the new strain on the LB agar plate were examined. Salt tolerance of the strain was examined by growth on LB medium supplemented with different salinity conditions [0.5–4.0% (w/v) at intervals of 0.5%].

For cellular fatty acid composition analysis, strains were aerobically cultivated on agar plate medium (per litre: 30 g of BBL Trypticase soy broth and 15 g of Difco granulated agar) at 28 °C for 2 days. Fatty acids were extracted from freeze-dried cells according to the protocol of the Sherlock Microbial Identification System (Version 6.0, MIDI, USA). The extracted fatty acids were analysed using GC and identified using the TSBA6 library.

For cellular respiratory quinone analysis, strains were aerobically cultivated on the modified LB agar plate mentioned above at 28 °C for 24 h. For respiratory quinone analysis, total cellular lipids were extracted from freeze-dried cells using the Bligh–Dyer method [16], and quinones were separated and purified using a Sep-Pak plus silica column (Waters, USA), followed by analysis using the ACQUITY UPLC H-Class system (Waters, USA). Cellular fatty acid and quinone analysis were performed at Techno Suruga Laboratory Co., Ltd. (Shizuoka, Japan).

### Genome analysis

Genomic DNA was extracted from bacterial cultures using the Quick-DNA Fungal/Bacterial Miniprep Kit (Zymo Research, USA), following the manufacturer’s protocol. The fragment size distribution of genomic DNA was assessed with the Genomic DNA 165 kb Kit (Agilent Technologies, USA). The library was constructed using the SMRTbell Prep Kit 3.0 (PacBio, USA), following the manufacturer’s guidelines for whole-genome sequencing. Sequencing was performed on the PacBio Revio platform (PacBio, USA) using Revio polymerase kits, sequencing plates and SMRT Cell trays at bitBiome Inc. (Tokyo, Japan).

The raw reads obtained from the PacBio Revio platform were assembled using Flye (v2.9.4) [17]. Contigs shorter than 1,000 bp were removed using Seqkit (v2.6.1) [18]. Assembly quality check and genome annotation were conducted using the DFAST web-based pipeline (https://dfast.ddbj.nig.ac.jp/) [19]. The completeness and contamination of the assembled genomes were assessed using CheckM (v1.1.3) [20]. Taxonomic classification was performed using GTDB-Tk (v2.3.2) [21] with the GTDB database release 214. Average nucleotide identity (ANI) values were calculated using AutoMLST2 [22]. The top 50 micro-organisms with the highest ANI values were used for digital DNA–DNA hybridization (dDDH) analysis using Genome-to-Genome Distance Calculator 3.0 [23]. Average amino acid identity (AAI) values were calculated using EzAAI [24]. Percentage of Conserved Proteins (POCP) values were calculated using the POCP-nf pipeline with default settings [25].

A genome-based phylogenetic analysis was performed using the autoMLST2.0: Automated Multi-Locus Species Tree pipeline (https://automlst2.ziemertlab.com/index) with *de novo* mode. This autoMLST2.0 analysis was performed based on alignment of conserved core genes present in closely related genomes from the NCBI Prokaryotic Genome Annotation Pipeline (https://doi.org/10.1093/nar/gkaa1105) database. For phylogenetic tree construction, the coalescent approach was selected, and ASTRAL-PRO3 was employed to estimate the species tree (https://doi.org/10.1093/molbev/msaa139). The final tree was visualized using iTOL (https://itol.embl.de/) [26].

## Results and discussion

### Morphology, physiology and biochemical characteristics

Strains HTMS2 and HTMS3^T^ were found to be Gram-stain-negative, non-spore-forming, non-motile rods, with ~0.6–0.8×1.0–2.0 µm. Colonies grown on LB agar plate were circular, convex (lens-shaped), smooth, cream-coloured and ~1–2 mm in diameter after 48 h of incubation. Both strains were catalase- and oxidase-positive, produced acid but no gas from glucose and exhibited both oxidative and fermentative glucose metabolism. Growth occurred at 37 °C but not at 45 °C, and anaerobic growth was positive. The major respiratory quinone identified in both strains was ubiquinone Q-8. Detailed results of the phenotypic and biochemical analyses of strains HTMS2 and HTMS3^T^ are provided in Table 1 and in the species description.

**Table 1.** Differential characteristics of strains HTMS2 and HTMS3^T^ and other phylogenetically related genera in the family *Burkholderiaceae* Strains: 1, strain HTMS2; 2, strain HTMS3^T^; 3, *Hydromonas duriensis* A2P5^T^ (data from Vaz-Moreira *et al*. [8]); 4, *Formosimonas limnophila* AHQ-12^T^ (Chen *et al*. [7]); 5, *Ephemeroptericola cinctiostellae* F02^T^ (Kim *et al*. [9]).

Characteristic	1	2	3	4	5
Cell size (µm)	0.6–0.8×1.0–2.0	0.6–0.8×1.0–2.0	0.7–2.2	0.3–0.4×0.6–1.2	0.4–0.5×0.7–2.8
Growth temperature range (°C)	28–37	28–37	10–30	10–37	4–30
NaCl tolerance (%)	0–1.5	0–1.5	0–1	0–1	0–0.5
pH range	7–9	7–9	6.0–8.0	5.0–9.0	5.5–8.5
Nitrate reduction	+	+	−	−	−
**Hydrolysis of:**					
Aesculin	−	−	−	−	+
**Assimilation of:**					
d-Mannitol	−	−	+	+	NR
d-Turanose	+	+	−	−	NR
Sorbitol	+	−	−	+	NR
Fructose	+	+	−	+	NR
Mannose	−	−	−	+	NR
Maltose	+	+	−	−	NR
Sucrose	+	+	−	−	NR
Trehalose	+	+	−	+	NR
*N*-Acetyl-glucosamine	−	−	+	+	+
Malic acid	+	+	−	−	+
Glycerol	+	+	−	−	NR
Ribose	+	+	−	−	NR
Gluconate	+	+	−	−	NR
**Enzymes:**					
Alkaline phosphatase	+	+	+	w	−
Leucine arylamidase	+	+	−	w	w
β-Galactosidase	+	+	+	−	+
α-Glucosidase	+	+	−	−	w
β-Glucosidase	−	−	−	−	+
Major quinone	Q-8	Q-8	Q-8	Q-8, Q-10	Q-8
Major fatty acids (>10%)	C16:1 ω7c/ω6c, C18:1 ω7c/ω6c, C16:0	C16:1 ω7c/ω6c, C16:0, C18:1 ω7c/ω6c	C16:1 ω7c/ω6c, iso-C15:0 3-OH, iso-C11:0	C16:1 ω7c/ω6c, iso-C15:0 3-OH, iso-C13:0 3-OH, iso-C11:0	C16:1 ω7c/ω6c, iso-C13:0 3-OH
DNA G+C content (mol%)	48.2	48.2	47	50.4	48.3

NR, not reported; W, weak positive reaction.

Several phenotypic and chemotaxonomic characteristics distinguished strains HTMS2 and HTMS3^T^ from related taxa (Table 1). Both strains were positive for nitrate reduction, while their closely related species *H. duriensis* A2P5^T^, *F. limnophila* AHQ-12^T^ and *E. cinctiostellae* F02^T^ did not show this reaction. Additionally, HTMS2 and HTMS3^T^ could utilize a broader range of carbohydrates than the related strains. In contrast, neither HTMS2 nor HTMS3^T^ could utilize *N*-acetyl-d-glucosamine, which is different from several other members of the family *Burkholderiaceae*. Although HTMS2 and HTMS3^T^ shared most phenotypic traits, they differed in sorbitol assimilation. Both strains had major fatty acids including summed feature 3 (C16:1 ω7c/ω6c), summed feature 8 (C18:1 ω7c/ω6c) and C16:0 at levels exceeding 10%. However, the relative abundances differed, with HTMS2 containing more summed feature 8 and HTMS3^T^ showing higher levels of C16:0. The fatty acid profiles of the strains differed markedly from that of closely related species of *H. duriensis* A2P5^T^, *E. cinctiostellae* F02^T^ and *F. limnophila* AHQ-12^T^, which contains iso-branched hydroxyl fatty acids such as iso-C15:0 3-OH and iso-C13:0 3-OH (Table 1). In contrast, both HTMS2 and HTMS3^T^ lacked these components and mainly possessed straight-chain unsaturated fatty acids.

### Phylogenetic analysis

**Fig. 1.**
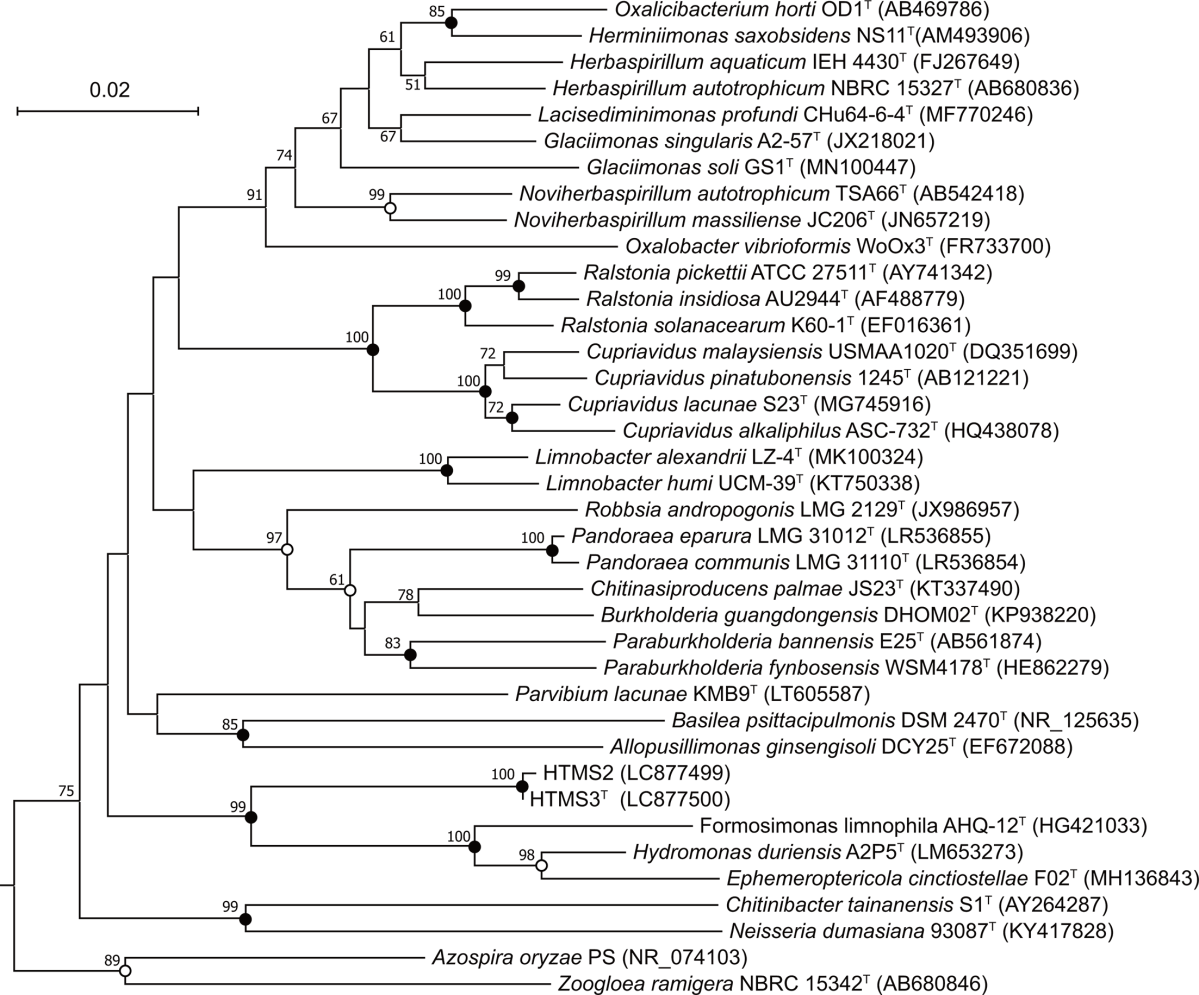
Phylogenetic position of strains HTMS2, HTMS3^T^ and representatives of other related taxa. The tree was constructed based on a distance matrix analysis of the 16S rRNA gene sequence using the neighbour-joining method. *Aquicella lusitana* SGT-39T (AY359282) was used as an outgroup. Bootstrap resampling analysis was performed for the neighbour-joining, maximum-likelihood and maximum-parsimony methods to estimate the confidence of the tree topologies. The numbers at the nodes indicate bootstrap values for the neighbour-joining method. Branching points with support above 50% in all three analyses are indicated by solid circles, whereas nodes with open circles indicate bootstrap support in the two analyses. The scale bar represents the 0.02 substitutions per nucleotide position.

The 16S rRNA gene sequence of strain HTMS3^T^ showed the highest similarity to *H. duriensis* A2P5^T^ (94.14%), followed by *E. cinctiostellae* F02^T^ (92.58%) and *F. limnophila* AHQ-12^T^ (92.38%). A 16S rRNA gene sequence identity threshold of ≤94.5% has been proposed to delineate distinct genera [27], which indicates that strain HTMS3ᵀ represents a novel genus within the family *Burkholderiaceae*. A phylogenetic tree based on 16S rRNA genes was reconstructed using the neighbour-joining method (Fig. 1), clearly indicating that strains HTMS2 and HTMS3ᵀ formed a distinct lineage within the family *Burkholderiaceae*, separate from existing genera. Bootstrap support values above 50% in neighbour-joining, maximum-likelihood and maximum-parsimony analyses are shown at nodes, confirming the phylogenetic distinction of strains HTMS2 and HTMS3ᵀ from related taxa.

**Fig. 2.**
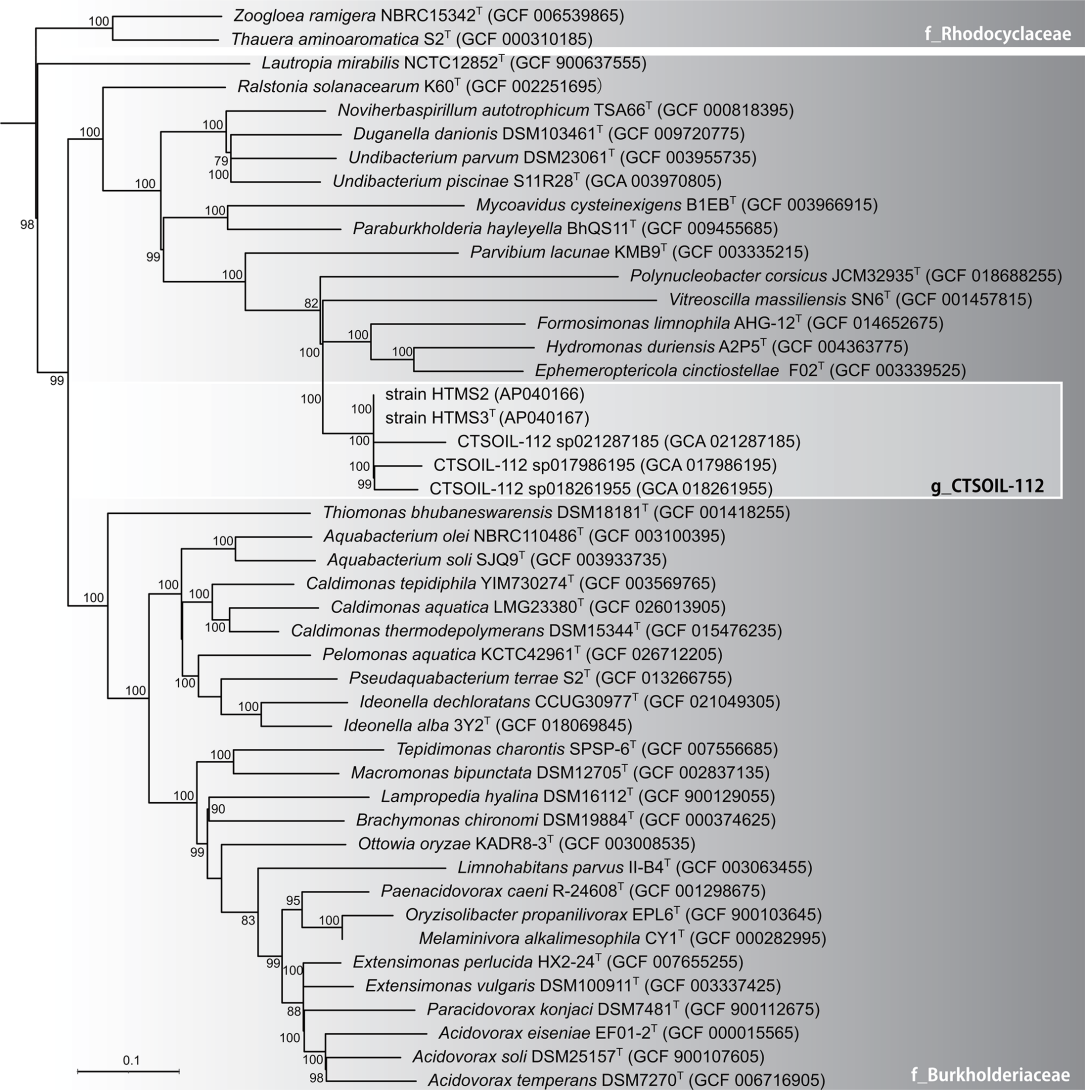
Maximum-likelihood phylogenomic tree based on conserved core genes indicating the phylogenetic position of strains HTMS2 and HTMS3^T^ with related species of family *Burkholderiaceae*. *Pseudomonas oryzicola* RD9SR1T (GCF_014269185) was used as an outgroup. The phylogenetic groups are according to GTDB (10). Bootstrap values (>75%) are shown at the nodes. Bar, 0.1 substitutions per nucleotide position.

The whole-genome-based phylogenetic tree (Fig. 2) placed strains HTMS2 and HTMS3ᵀ within the family *Burkholderiaceae*, but in a distinct lineage separate from genera *Hydromonas* and *Formosimonas*. The strain HTMS3ᵀ formed a robust clade with representative strains of the uncultured genus-level lineage named CTSOIL-112 (bootstrap=100%) based on the classification of the GTDB database release 226 (https://gtdb.ecogenomic.org/taxon-history?from=R95&to=R226&query=g__CTSOIL-112). These results strongly support that HTMS3ᵀ represents the first representative of a previously uncultured genus CTSOIL-112.

### Genomic features

Nearly complete genomes were obtained, and the genomes of strains HTMS2 and HTMS3ᵀ were highly similar, with genome sizes of ~2.52 Mbp, high completeness (99.43%) and low contamination (0.03%). The ANI and dDDH values between strains HTMS2 and HTMS3ᵀ are 99.9% and 100%, respectively; thus, further genomic analysis was conducted only on strain HTMS3ᵀ. The ANI and dDDH values between strain HTMS3ᵀ and related strains are shown in Table S1, available in the online Supplementary Material. The ANI values are all below the species boundary threshold of 95–96% [28], with the highest ANI being 77.0% to CTSOIL-112 sp017986195, and the highest ANI to an isolated strain was *Moraxella osloensis* YV1 with 74.9%. Similarly, dDDH values were below the 70% threshold typically used for species boundary, with the highest dDDH value of 54.9% to *Aeromonas* sp. strain 3925. In addition to ANI and dDDH analyses, AAI and POCP were calculated to evaluate genus-level relationships. The AAI values between strain HTMS3ᵀ and closely related species of *H. duriensis* A2P5^T^, *E. cinctiostellae* F02^T^ and *F. limnophila* AHQ-12^T^ were 68.3–68.6%, while those to the GTDB lineage CTSOIL-112 were 76.7–76.8% (Table S2). Correspondingly, POCP values between HTMS3^T^ and these genera were 57.9–58.9%, whereas those to CTSOIL-112 MAGs were 58.5–67.0% (Fig. S1). These results indicate that there is a clear difference between the described genera (e.g. *Hydromonas* and *Formosimonas*) and the GTDB lineage CTSOIL-112 in the values of AAI and POCP, and AAI values could discriminate the genera [29]. These results support the recognition of HTMS3ᵀ as a novel species within a novel genus in the family *Burkholderiaceae*.

KEGG pathway analysis revealed that strain HTMS3ᵀ possesses complete modules for central carbohydrate metabolism, including glycolysis, gluconeogenesis and the tricarboxylic acid cycle, suggesting versatile carbon utilization. Consistent with biochemical analysis results, the strain HTMS3ᵀ possesses nitrate reductase genes, such as *narG*, but lacks nitrite reductase genes such as *nirK*, *nirB* and *nasD*. Therefore, the strain is predicted to reduce nitrate only to nitrite, without further denitrification or dissimilatory/assimilatory nitrate reduction.

In accordance with physiological analysis results, the genome of strain HTMS3ᵀ lacks motility-related genes, and only *motA* was detected, whereas those such as *motB*, *cheW* and *fliC* were absent. It has been experimentally demonstrated that the deletion of genes such as *fliC* or *motA* results in a complete loss of motility [30]. Additionally, outer membrane protein and lipopolysaccharide biosynthesis-related genes such as *lptD* and *bamC* were detected [31], and genes specific to Gram-positive bacteria involved in teichoic acid biosynthesis (e.g. *tag*, *dlt* and *ltaS*) were not detected. The three metagenome-assembled genome (MAG) sequences registered as g_CTSOIL-112 in GTDB, with more than 80% of completeness, were also analysed. The analysis results of MAGs GCA_018261955.1, GCA_021286655.1 and GCA_021287185.1 showed that these MAGs lack most of the motility-related genes and also have no genes specific to Gram-positive bacteria mentioned above. Moreover, multiple genes related to the outer membrane and lipopolysaccharide components were detected. Based on these results, g_CTSOIL-112 is considered a non-motile Gram-negative genus.

Strains HTMS2 and HTMS3^T^ were isolated as independent colonies from the same biofilm sample and showed slightly different phenotypic characteristics, such as sorbitol assimilation and relative fatty acid composition. However, the two strains showed 99.86% of 16S rRNA gene sequence identities, and the whole-genome comparison revealed 99.9% ANI and 98.5% dDDH between them. Therefore, HTMS2 and HTMS3^T^ are regarded as different strains belonging to the same species.

Based on the above results, phylogenetic and genomic analysis clearly indicated that strain HTMS3^T^ represents a new species and a previously uncultured genus CTSOIL-112 based on GTDB within the family *Burkholderiaceae*. Based on this, the name *P. membranae* gen. nov., sp. nov. is proposed.

## Description of *Puribacter* gen. nov.

*Puribacter* [Pu.ri.bac′ter. L. masc. adj. *purus*, clean; N.L. masc. n. *bacter*, a rod; N.L. masc. n. *Puribacter*, a rod-shaped bacterium isolated from wastewater treatment sludge (activated sludge)].

Cells are Gram-staining-negative rods. Oxidase and catalase are positive. The predominant quinone is ubiquinone Q-8. Major cellular fatty acids (>10%) are summed feature 3 (C16:1 ω7c/ω6c), summed feature 8 (C18:1 ω7c/ω6c) and C16:0. The DNA G+C content of the type strain of the type species is 48.2 mol%. The type species is *P. membranae*.

## Description of *Puribacter membranae* sp. nov.

*Puribacter membranae* (mem.bra′nae. L. gen. n. *membranae*, of a membrane, referring to the isolation source of the type strain from a membrane biofilm of a sewage treatment bioreactor).

Cells are Gram-stain-negative, non-spore-forming, non-motile rods, ~0.6–0.8 µm wide and 1.0–2.0 µm long. Colonies on LB agar are circular, convex (lens-shaped), smooth, cream-coloured and ~1–2 mm in diameter after 48 h of incubation at 30 °C. Growth occurs at 28–37 °C and pH 7.0–9.0. Growth is observed under both aerobic and anaerobic conditions. Catalase and oxidase reactions are positive. Acid or gas production from glucose fermentation is observed, and oxidation-fermentation tests of glucose are both positive. In API 20 NE, API 20 E and API 50CH tests, assimilation of glucose, maltose, galactose, fructose, sucrose, trehalose, d-turanose, gluconate, dl-malic acid, glycerol and ribose is observed. In the API 20 NE, API 20E, and API ZYM tests, positive enzymatic reactions were observed for nitrate reduction, β-galactosidase, cytochrome oxidase, oxidase, nitrite production, alkaline phosphatase, C4 esterase, leucine arylamidase, acid phosphatase, and α-glucosidase, with weakly positive reactions for C8 esterase lipase and naphthol-AS-BI-phosphohydrolase. Major cellular fatty acids (>10%) are summed feature 3 (C16:1 ω7c/ω6c), summed feature 8 (C18:1 ω7c/ω6c) and C16:0. The quinone system is ubiquinone Q-8. The DNA G+C content of the type strain is 48.2 mol%. The type strain HTMS3^T^ (NBRC 117344^T^=LMG 34133^T^) was isolated from a sewage treatment MBR at the Nagaoka Central Sewage Treatment Center, Nagaoka City, Niigata Prefecture, Japan (37° 28′ 55′ N 138° 51′ 16′ E). The GenBank/EMBL/DDBJ accession numbers for the 16S rRNA gene sequence and the nearly complete genome sequence of strain HTMS3^T^ are LC877500 and AP040167.

## Supplementary material

10.1099/ijsem.0.006978 Uncited Fig. S1.

10.1099/ijsem.0.006978 Uncited Fig. S2.

## Abbreviations

AAI: average amino acid identity
ANI: average nucleotide identity
dDDH: digital DNA–DNA hybridization
GTDB: Genome Taxonomy Database
MAG: metagenome-assembled genome
MBRs: membrane bioreactors
NCBI: National Center for Biotechnology Information
POCP: Percentage of Conserved Proteins
