# Supplementary material for: Puribacter membranae gen. nov., sp. nov., isolated from a biofilm of a membrane bioreactor (MBR) treating sewage
Source: Int J Syst Evol Microbiol. 2025 Nov 20;75(11):006978. doi: 10.1099/ijsem.0.006978 (PMC12634028; doi:10.1099/ijsem.0.006978)
Supplement: Uncited Fig. S1. [file ijsem-75-06978-s001.pdf]

Supplementary materials

Puribacter membranae gen. nov., sp. nov., isolated from a biofilm of membrane bioreactor (MBR) treating sewage

Masashi Hatamoto[1]*

1 Department of Civil and Environmental Engineering, Nagaoka University of Technology, Niigata 940-2188, Japan

* Corresponding author

Department of Civil and Environmental Engineering, Nagaoka University of Technology, 1603-1 Kamitomioka, Nagaoka, Niigata 940-2188, Japan

Tel: +81-258-47-9637, Fax: +81-258-47-9637

E-mail address: hatamoto@vos.nagaokaut.ac.jp (Masashi Hatamoto)

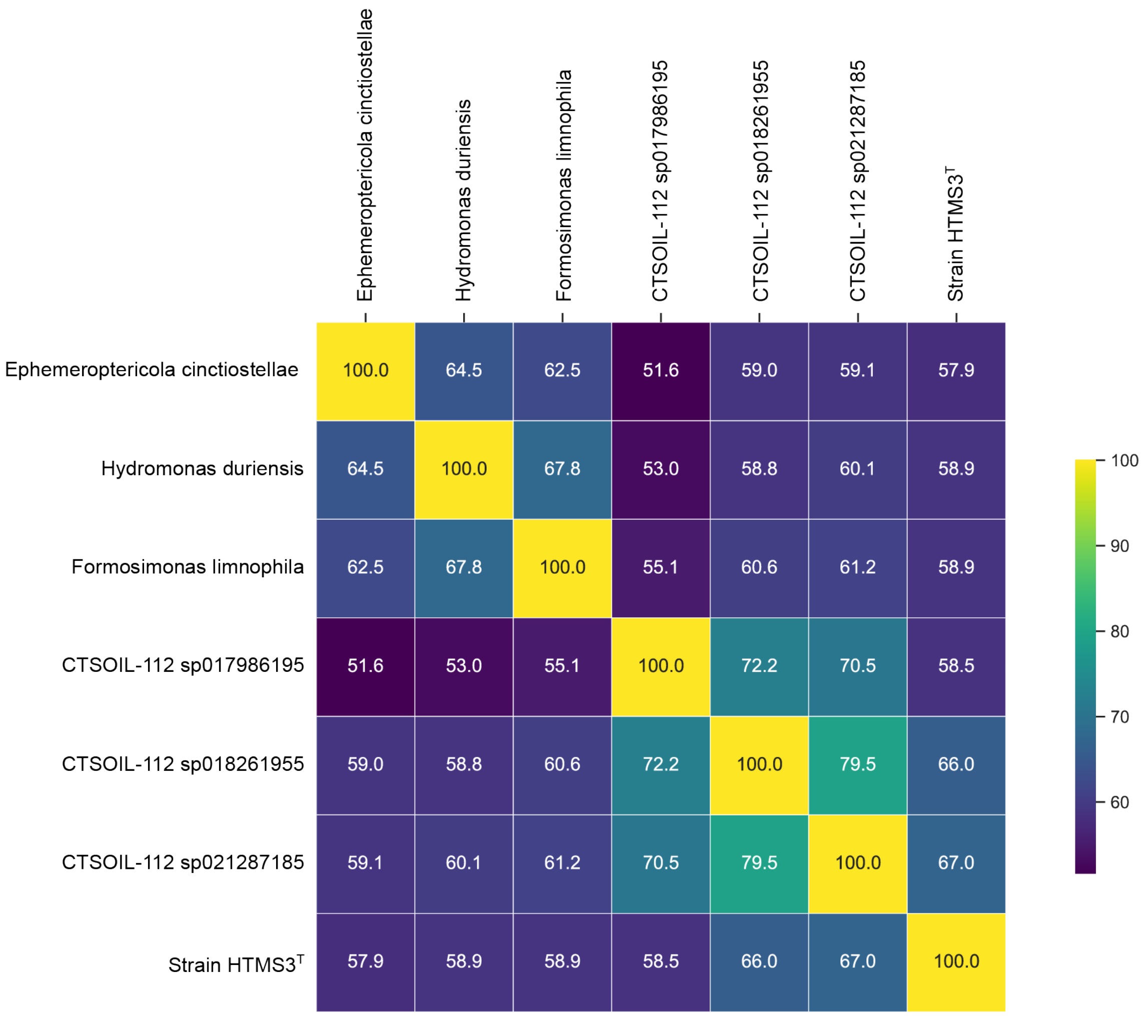

Fig.S1 Percentage of conserved proteins (POCP) for the strain HTMS3^T genome compared with closely related species.
